# Body Composition and Its Impact on the Hormonal Disturbances in Women with Polycystic Ovary Syndrome

**DOI:** 10.3390/nu13124217

**Published:** 2021-11-24

**Authors:** Anna Bizoń, Sylwia Płaczkowska, Justyna Niepsuj, Marta Czwojdzińska, Marcin Leśniewski, Artur Nowak, Dagmara Pluta, Paweł Madej, Agnieszka Piwowar, Grzegorz Franik

**Affiliations:** 1 Department of Toxicology, Faculty of Pharmacy, Wroclaw Medical University, 50-367 Wroclaw, Poland; marta.czwojdzinska@umw.edu.pl (M.C.); agnieszka.piwowar@umw.edu.pl (A.P.); 2 Department of Laboratory Diagnostics, Diagnostics Laboratory for Teaching and Research, Faculty of Pharmacy, Wroclaw Medical University, 50-556 Wroclaw, Poland; sylwia.placzkowska@umw.edu.pl; 3 Students Scientific Society at the Department of Toxicology, Faculty of Pharmacy, Wroclaw Medical University, 50-556 Wroclaw, Poland; justyna.niepsuj@student.umw.edu.pl; 4 Department of Gynaecological and Obstetrics, District Hospital, 43-200 Pszczyna, Poland; marcinlesniewski@wp.pl; 5 Gynecological and Obstetrician Polyclinic, District Hospital, 15-435 Białystok, Poland; artur_nowak@mp.pl; 6 Department of Endocrinological Gynecology, Medical University of Silesia, 40-055 Katowice, Poland; dpluta@sum.edu.pl (D.P.); pmadej@sum.edu.pl (P.M.); antoni.franik@gmail.com (G.F.)

**Keywords:** polycystic ovary syndrome, body composition, hormones

## Abstract

We investigated the relationship between selected body composition (BC) parameters and included 55 women diagnosed with PCOS and 29 women in which PCOS was excluded. Hormone concentration and BC parameters were assessed during hospitalization. Women with PCOS had higher concentration of luteinizing hormones, total testosterone, androstenedione, and Anti-Müllerian hormones compared to women that were not diagnosed with PCOS. We did not observe any significant differences in the BC parameters between both groups as well as between four PCOS phenotype subgroups. Only in the group of women with PCOS was the concentration of sex hormone binding globulin and free testosterone correlated with all investigated BC parameters. Correspondence analysis did not confirm unambiguously associations between phenotypes of PCOS and the value of BC parameters, while logistic regression revealed that increased Anti-Müllerian hormone concentration and the value of body mass index could be useful parameters in differentiating women with PCOS and women with other disorders. The ROC analysis performed on the entire group of women also confirmed that the concentration of Anti-Müllerian hormones could be a powerful parameter to categorize women as suffering from PCOS.

## 1. Introduction

Improper diet, abnormal body weight, and lack of physical activity are some of the crucial factors associated with development of many diseases as well as their exacerbation [1]. Polycystic ovary syndrome (PCOS) is a common reproductive and endocrine disorder in which obesity intensifies metabolic and hormonal disorders [2]. According to studies 30–70% of women with PCOS are obese [3] and many are characterized by abdominal obesity [4], which contributes to the clinical and biochemical expression of PCOS [5,6]. Weight reduction for obese patients with PCOS is beneficial in many ways. It helps decrease the concentration of androgen, luteinizing hormone and insulin as well as regulate ovulation [7], therefore lifestyle improvement is the first line of treatment for women with PCOS [8]. As the primary cause of PCOS is still unknown, the treatment is primarily directed at symptoms [7]. Diagnosis of PCOS is based on meeting at least two out of three Rotterdam criteria: clinical or/and biochemical hyperandrogenism, ovulatory dysfunction, and polycystic ovaries found on an ultrasound [9]. According to these criteria it is possible to identify four PCOS phenotypes: (1) with clinical and/or biochemical hyperandrogenism, oligoovulation or anovulation and polycystic ovaries; (2) with clinical and/or biochemical hyperandrogenism and oligoovulation or anovulation; (3) with clinical and/or biochemical hyperandrogenism and polycystic ovaries; and (4) with oligoovulation or anovulation and polycystic ovaries [10]. However, studies show no significant differences in prevalence of insulin resistance and metabolic syndrome in different PCOS phenotypes [11]. The most widely used parameters which help to diagnose obesity are body mass index (BMI), waist circumference (WC), waist-to-hip ratio (WHR), visceral fat area (VFA), and body fat percentage (BFP) [12]. Considering the presence of sexual dimorphism in fat distribution, we already know that androgens play an important role in distribution of adipose tissue in the human body [13]. Several previous studies showed a correlation between androgen levels and amount of abdominal fat and visceral adipose tissue [14,15]. It was also demonstrated that women with abdominal obesity have lower concentrations of sex hormone bounding globulin (SHBG), which supports the thesis that free androgens are related to increased visceral fat accumulation [14]. Furthermore, earlier studies also pointed out that android type of fat distribution is associated with reduced reproductive capability and infertility [16]. Our study showed that abdominal obesity causes additional disorders in metabolic and hormonal parameters in PCOS women [17], suggesting that further investigation is needed to evaluate the influence of body fat distribution on PCOS to be able to improve the course of future treatment. Our previous study [18] and the results obtained by other authors of [19] indicate that the value of BMI and WC could be insufficient to measure the percentage of body fat. Furthermore, the value of BMI does not capture information on the mass of fat in different body sites and cannot diversify central and peripheral fat [20,21]. Analyzing body composition is much more useful than measuring body weight, since it can distinguish body fat mass from fat free mass [22]. In our previous study we proposed bioelectric impedance as a minimally invasive method that can be recommended for the assessment of body weight, especially in young people [23]. Therefore, in our present study we aimed to investigate the relationships between selected body composition parameters and hormones concentration (follicle-stimulating hormone (FSH), luteinizing hormone (LH), total testosterone (*t*-test), free testostereone (fTest), androstenedione (AD), dehydroepiandrosterone sulfate (DHEA-S), SHBG, 17α-hydroxyprogesterone (17α-OHP), and Anti-Müllerian hormones (AMH)) in a group of women with and without PCOS. We used bioelectric impedance analysis (Bodystat 1500), which is a non-invasive, reliable, low-cost, easy to use, and clinically validated method.

## 2. Materials and Methods

The study was conducted on the group of 84 women who were hospitalized in the Gynecological Endocrinology Clinic of the Silesian Medical University in Katowice, Poland, in 2021. On average, 90 ± 10 women suspected of PCOS are hospitalized at the clinic every month. We selected a case-control study of women aged 18–43, who were hospitalized in April 2020. According to Rotterdam criteria, 55 women were diagnosed with PCOS (study group) and 29 women were excluded as not suffering from PCOS (control group). The study was approved by the Bioethical Committee of the Medical University of Silesia (No KNW/0022/KB1/140/II/15/16, 15 November 2016). In addition, in the group of women with PCOS, four different phenotypes of PCOS were recognized based on Rotterdam criteria. In the group of women without PCOS, one woman had polycystic ovaries, six women had oligomenorrhea, and seven women were characterized by high levels of androgens, but none of them were diagnosed with PCOS based on Rotterdam criteria. In both groups, women exposed to tobacco smoke or alcohol abuse were excluded from the study. Other exclusion criteria included previously diagnosed diabetes (type 1 and 2), hypertension, Cushing’s syndrome, and adrenal tumors. Blood samples were collected during the follicular phase (till day eight of the menstrual cycle) in the morning after an overnight fast (>12 h) according to standard procedures. Blood samples were immediately centrifuged and serum and plasma were separated; parameters associated with PCOS were assayed immediately.

### 2.1. Assessment of Body Composition

During hospitalization in all women body composition parameters were assessed based on bioimpedance methods using Bodystat 1500 (Douglas, Isle of Man) according to the instructions provided by the producer. The participants were asked not to eat for 4–5 h and not to exercise for 12 h before the test. The Bodystat 1500 was calibrated at the beginning of a day of use. We focused on selected body composition parameters including body mass index (BMI) (kg/m^2^); waist hip ratio (WHR); fat-free mass (FFM); body weight (BW) (kg); skeletal muscle mass (SMM) (kg); fat mass (FAT) (kg); total body water (TBW) (%); percent of body fat (PBF) (%); visceral fat area (VFA) (cm^2^); and basal metabolic rate (BMR) (kcal).

### 2.2. Hormone and SHBG Concentrations

FSH, LH, *t*-test, fTest, AD, DHEA-S and SHBG concentrations were determined by ELISA (DRG Instruments GmbH, Marburg, Germany) with the lower limit of sensitivity at 0.86 IU/L, 1.27 IU/L, 0.083 µg/L, 0.002 ng/L, 0.019 µg/L, 0.044 mg/L and 0.2 nmol/L, respectively; the respective intra- and inter-assay coefficients of variations were 5.5% and 6.1% for FSH, 5.6% and 6.2% for LH, 3.6% and 7.1% for total testosterone, 6.4% and 8.0% for free testosterone, 6.5% and 10.2% for androstenedione, 4.8% and 7.5% for DHEA-S and 5.3% and 9.0% for SHBG. 17α-hydroxyprogesterone (17α-OHP) was assayed by RIA (Diagnostic Products Corporation, Los Angeles, CA, USA) with lower detectable concentrations of 0.2 nmol/L. The respective inter- and intraassay coefficients of variation were 5.6% and 8.0% for 17-OH-P. The concentration of AMH was assayed using commercial ELISA kit (Immunotech a.s., Prague, Czech Republic) with lower detectable concentrations of 0.05 ng/mL, inter- and intra- assay coefficient of variations were 4.6% and 4.6%, respectively.

### 2.3. Statistical Analysis

Statistical analysis was performed using the Statistica Software Package, version 13.3 (Polish version; StatSoft, Kraków, Poland). Values, except age (presented as mean ± SD) were expressed as median with interquartile range (IQR). Normality of variables was tested using the Shapiro-Wilk test. Homogeneity of variance was assessed using Levene’s test. Significant differences in hormones concentration and body composition parameters between groups of women with and without PCOS were investigated using non-parametric U Mann-Whitney test. Differences between four PCOS phenotype subgroups were analyzed using Kruskal-Wallis one-way analysis of variance by ranks. The correspondence analysis was performed to evaluate the potential association between quartile of body composition parameters and phenotypes of PCOS. Logistic regression was used to indicate which of the analyzed parameters increased the patient’s odds of belonging to the PCOS group. Furthermore, the ROC (receiver operating characteristic) analysis was performed to show which parameter best categorized women as suffering from PCOS. In all instances, *p* < 0.05 was considered statistically significant.

## 3. Results

The studied groups were similar in terms of age. Women with PCOS had higher concentration of LH, *t*-test, AD, and AMH compared to women without PCOS. The median of AMH concentration was two-fold higher in the group of women with PCOS than in the group of women without PCOS. Any other significant differences in hormones concentration and selected values of body composition parameters were not found (Table 1).

Comparison of the value of body composition between four phenotypes of PCOS did not show any statistically significant differences (Table 2).

Separately, in both study and control groups we investigated the relationships between hormones concentrations and selected body composition parameters. In the group of women with PCOS, a negative correlation between the concentration of SHBG and the value of selected body composition parameters was found, while a positive correlation was revealed in case of the concentration of fTest and the value of some body composition parameters (Table 3). In the control group, the concentration of SHBG did not correlate with any parameters of body composition, while the concentration of fTest was only associated with PBF value (r = 0.40; *p* = 0.035). Only in the control group we found a positive correlation between the concentration of DHEA-S and the value of BMI (r = 0.39; *p* = 0.038) as well as a negative one between the concentration of 17-OHP and the value of WHR (r = −0.47; *p* = 0.010) (results not shown in the table).

**Figure 1:**
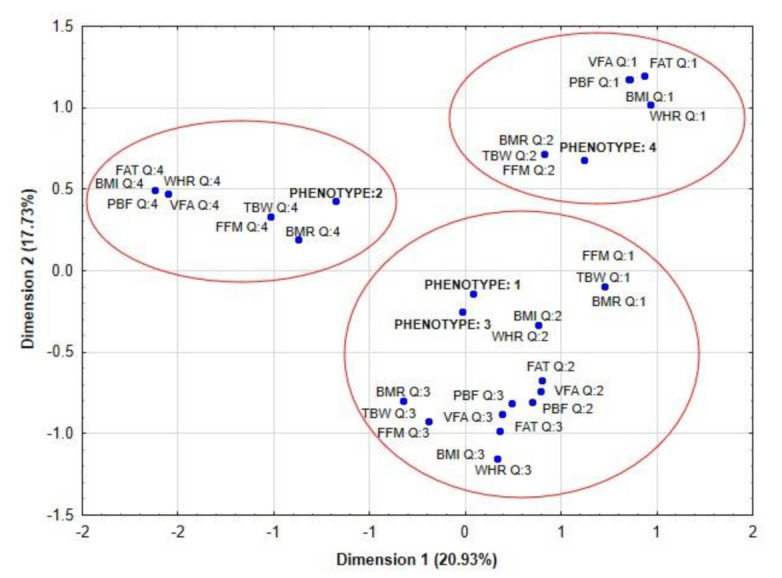
Correspondence analysis showed the potential association between quartiles of body composition parameters and phenotypes of PCOS. Legend: PCOS, polycystic ovary syndrome; BMI, body mass index; WHR, waist hip ratio; FAT, fat mass; TBW, total body water; FFM, fat-free mass; PBF, percent of body fat; VFA, visceral fat area; BMR, basal metabolic rate.

Correspondence analysis showed that phenotype 2 of PCOS was associated with the highest value (assigned to Q4) of analyzed body composition parameters (FAT, BMI, PBF, VFA, FFM, BMR), while phenotype 4 was related to the lowest value (assigned to Q1 or 2) of body composition parameters (for Q1: VFA, FAT, BMI, WHR, PBF and for Q2: TBW, BMR, FFM). Women with phenotype 1 and 3 presented average values of body composition (Figure 1).

Logistic regression revealed that increased concentration of AMH and selected values of body composition such as BMI, PBF, and VFA analyzed as a separated models could be useful in differentiating women with PCOS and women with other disorders, which are not associated with PCOS (Table 4). Model 4 revealed that among body composition parameters analyzed separately: BMI, PBF and VFA, BMI was the strongest and independent (other than AMH) variable related to increased odds of belonging to the PCOS group.

**Figure 2:**
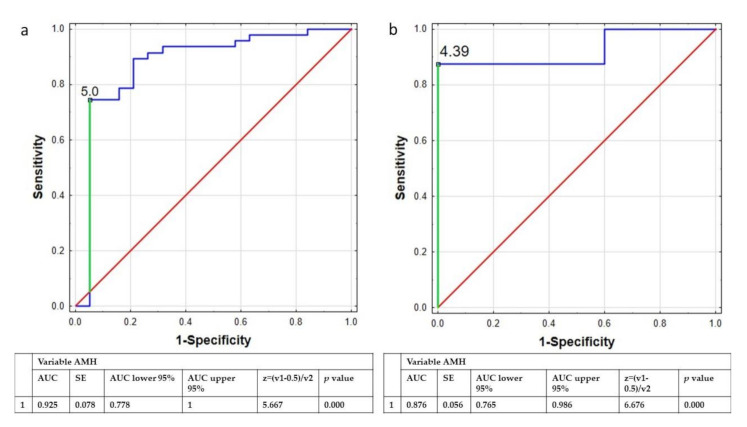
ROC curve of AMH concentration used to distinguish the women with PCOS from the whole group of women younger (**a**) and older than 30 years old (**b**). Legend: ROC curve, receiver operating characteristic curve; AMH, Anti-Müllerian hormone; AUC, area under curve; SE, sedimentation equilibrium.

Both ROC analysis performed in the group of women <30 years old and >30 years old indicated that specific concentrations of AMH could be a powerful parameter to categorize women with PCOS. The cut-off concentration of AMH for women <30 years was estimated as 5.00 ng/mL, while in the group of women >30 years old the threshold value was 4.39 ng/mL (Figure 2).

## 4. Discussion

The distribution of fat tissue in the visceral area is associated with many metabolic and endocrine disturbances in PCOS [24]. It seems that obesity plays a fundamental role in pathogenesis of PCOS [25]. Furthermore, Della Corte, et al. claimed that the main goal of therapy in PCOS should be the reduction of body mass [26]. Therefore, in our present study we were focused on the potential associations between the value of selected body composition parameters and the magnitude of hormonal disorders in women with PCOS. Firstly, we compared the results of selected body composition parameters between women with and without PCOS. We did not find any significant differences in the value of body composition parameters between those groups, which may be associated with a small number of patients and their similar physical constitution. The same findings were made when we analyzed those differences in four phenotypes of PCOS. Presumably the lack of statistically significant differences between four phenotypes of PCOS was also associated with a small number of cases, especially in phenotype 2 (*n* = 7) and 4 (*n* = 6). The correspondence analysis showed that body composition parameters were not directly associated with phenotypes of PCOS. Women with phenotype 2 were mainly associated with quartile 4 of BMI, PBF, and FFM values; regarding body composition parameters, women with phenotype 1 and 3 had average values of body composition parameters (e.g., the value of VFA, FFM and PBF), while women with phenotype 4 had mainly the lowest value of body composition parameters.

Our outcome indicates that the greatest disorders in body composition parameters are present in phenotype 2, which similar to the results obtained by Giampaolino, et al. could suggest that the women with phenotype 2 of PCOS has a different pathophysiologic mechanism of disease [27].

Using logistic regression analysis we showed, especially in Model 4, that among parameters analyzed separately the value of BMI was the strongest (besides AMH) variable related to the increased odds ratio of belonging to PCOS group (Table 4).

When we investigated the coefficient correlation between body composition parameters and hormones concentration in the group of women with PCOS we found a significant negative correlation between the concentration of SHBG and the value of all analyzed body composition parameters. The highest value of coefficient correlations was found between the concentration of SHBG and the value of BMI, WHR, FAT, and VFA, which showed clearly that the concentration of SHBG was associated with the amount and placement of fat tissue in visceral area (WHR and VFA). A study conducted by Azard, et al. [28] demonstrated that in the group of premenopausal women the serum concentration of SHBG was negatively associated with intra-abdominal adipose tissue. Moreover, a fat depot was linked with an abnormal metabolic profile as well as inversely and independently associated with SHBG [28]. Our earlier study showed that women with PCOS who were obese in the visceral area (WHR > 0.8) had lower concentration of SHBG and higher concentration of DHEA-S and value of FAI compared to women with PCOS and a normal value of WHR (<0.8) [17]. Furthermore, our last study revealed that disorders in hormonal status are probably indirectly associated with changes in lipid profile parameters [29]. Higher quartiles of triglycerides concentration increased the odds ratio of lower concentration of SHBG or higher value of free androgen index (FAI), while higher quartiles of HDL-C level decreased concentration of SHBG concentration and FAI value. Moreover, we also discovered that the phenotypes of PCOS, as well as the value of BMI, were significant factors which influenced androgen hormone concentrations [29].

Interestingly, in our present study in the group of women without PCOS the concentration of SHBG was not significantly associated with any parameters associated with body composition analysis. Similar results were previously obtained in the study conducted by Yucel et al. [30], who showed a negative correlation between the concentration of SHBG and the value of total fat mass in PCOS women. At the same time, there was no similar relationship in the group of healthy control. Apart of SHBG, in the women with PCOS fTest concentration was in positive relation to investigated body composition parameters, with the highest value of coefficient correlation with BMI (r = 0.47), WHR (r = 0.45), FAT (r = 0.42) and VFA (r = 0.41), while in the group of women without PCOS the concentration of free testosterone was only positively correlated with the value of PBF. Our study confirmed the results obtained by other authors, with lower concentration of SHBG being associated with an elevation in fTest concentration. Moreover, we confirmed that both of these parameters are closely related to obesity [31], which is in accordance with results obtained by other authors [32].

It was confirmed that SHBG could play an important role in various metabolic disorders in women with PCOS [33]. The meta-analysis conducted by Lim, et al. showed that overweight or obese women with PCOS had decreased concentration of SHBG and increased concentration of *t*-test and free androgen index [34]. Furthermore, hepatic synthesis of SHBG can be suppressed by insulin, while excess of insulin is common in the group of overweight women. It has been shown that insulin rises free and total testosterone, and this contributes to upper-half type body fat distribution [35]. The results obtained in our study confirmed that a decreased concentration of SHBG is associated with obesity especially distributed in the visceral area.

The second important parameter taken under consideration was the concentration of AMH. We observed a significant difference in AMH concentration between women with and without PCOS, but we did not detect any correlation with body composition parameters. Similar result was obtained by Bialka-Kosiec, et al. [36], who also did not discover any significant correlation between body mass components including body fat, fat-free mass, or water content and the concentration of AMH in women with PCOS. A study conducted by Cassar, et al. also did not reveal any significant correlation between the concentration of AMH and the value of body fat parameters [37]. The newest study conducted in the Chinese population revealed the negative correlation between body fat percentage and the concentration of AMH, and the authors claimed that the study should be continued to explain the association between body fat-related AMH change with pathogenesis of PCOS [38]. In addition, using ROC analysis the authors showed that AMH can be helpful in distinguish between women with and without PCOS (the value of AUC was 88.5%, with a sensitivity of 72.4% and specificity of 87.4%, the positive and negative predictive values were 91.2% and 63.9%, respectively. The same findings were demonstrated in a study conducted by Cassar, et al. [37]. Similarly, in our study we confirmed using the ROC analysis that concentration of AMH is a sensitive and specificity parameter associated with PCOS. We performed the ROC analysis in two groups of women: < and >30 years old, as the concentration of AMH changes with the age [39]. The value of AUC in both groups of women < and >30 years old was 87.6% and 92.5%, which confirmed that concentration of AMH reflects the probability of belonging to the PCOS group. Furthermore, the results obtained by Casarotto, et al. indicated that the concentration of AMH could be a prognostic parameter of PCOS in women with metabolic and hormonal disorders. AMH was associated with the pathophysiology of reproductive dysfunction in PCOS and reflects PCOS status, therefore it may also be useful in PCOS diagnosis [40]. Dadachanji, et al. [41] revealed the importance of clinicians concurrently employing Rotterdam criteria along with obesity status for ascertaining accurate disorders in the course of PCOS status.

Summarizing, in the group of women with PCOS the body composition parameters were mainly associated with the concentration of SHBG and fTest, while the parameters which are most strongly and independently related to belonging to the PCOS group were the concentration of AMH and the value of BMI.

Some limitations should be considered when interpreting these study results. Due to a small number of PCOS phenotype patients as well as of the control group, the obtained results should be treated as a pilot, and should be considered for future studies.

## 5. Conclusions

1. In the group of women with PCOS, the alteration in the value of body composition parameters were significantly associated with the concentration of SHBG and fTest.
2. The concentration of AMH and the value of BMI were the parameters most strongly and independently related to belonging to the PCOS group.

## Figures and Tables

**Table 1:** Characteristics of women with PCOS including selected body composition parameters.

Parameters	Women		
	without PCOS	with PCOS	*p* Value
Age (years)	28.24 ± 6.24	25.91 ± 4.70	0.058
LH (lU/L)	5.08 (4.17–6.55)	6.79 (5.23–9.36)	0.006
FSH (lU/L)	7.09 (5.89–8.21)	6.16 (5.27–7.39)	0.056
DHEA-S (µg/mL)	276.00 (216.00–350.00)	284.50 (217.00–384.00)	0.566
SHBG (nmol/L)	53.2 (37.00–75.10)	46.70 (30.70–73.10)	0.399
*t*-test (ng/mL)	0.22 (0.13–0.30)	0.28 (0.19–0.39)	0.025
fTest (pg/mL)	1.64 (0.93–3.02)	2.26 (1.28–3.39)	0.116
AD (ng/mL)	1.89 (1.37–2.26)	2.36 (1.81–3.13)	0.005
17α-OHP (nmol/L)	0.67 (0.39–0.92)	0.65 (0.46–0.89)	0.756
Prolactine (ng/mL)	9.81 (6.83–13.30)	11.60 (8.80–15.70)	0.086
AMH (ng/mL)	2.95 (1.58–3.63)	6.07 (4.84–8.25)	0.000
BMI (kg/m^2^)	20.90 (19.80–23.80)	24.00 (20.50–30.30)	0.069
WHR	0.85 (0.83–0.90)	0.87 (0.83–0.96)	0.168
SMM (kg)	23.40 (21.20–25.10)	24.70 (22.20–28.40)	0.094
FAT (kg)	17.80 (13.50–22.50)	21.60 (13.60–31.90)	0.157
TBW (%)	31.10 (28.50–33.30)	32.70 (29.70–37.60)	0.081
FFM (%)	42.60 (39.00–45.30)	44.70 (40.60–51.40)	0.082
PBF (%)	31.00 (39.00–45.30)	32.40 (24.20–39.00)	0.271
VFA (cm^2^)	8.00 (5.00–10.00)	9.00 (5.00–15.00)	0.236
BMR (kcal)	1290.0 (1212.0–1349.0)	1336.0 (1246.0–1479.0)	0.080

Legend: PCOS, polycystic ovary syndrome; LH, luteinizing hormone; FSH, follicle-stimulating hormone; DHEA-S, dehydroepiandrosterone sulfate; SHBG—sex hormone-binding globulin; *t*-test—total testosterone; fTest, free testosterone; AD, androstenedione; 17α-OHP, 17α-hydroxyprogesterone; AMH, Anti-Müllerian hormone; BMI, body mass index; WHR, waist hip ratio; SMM, skeletal muscle mass; FAT, fat mass; TBW, total body water; FFM, fat-free mass; PBF, percent of body fat; VFA, visceral fat area; BMR, basal metabolic rate.

**Table 2:** Comparison of the value of body composition parameters dependent on phenotype in the group of women with PCOS.

Parameters	Phenotype 1	Phenotype 2	Phenotype 3	Phenotype 4	*p* Value
	*n* = 31	*n* = 7	*n* = 11	*n* = 6	
Age (years)	26.00 (22.00–29.00)	25.000 (19.00–27.00)	25.00 (24.00–28.00)	27.50 (2500–33.00)	0.546
BMI (kg/m^2^)	23.40 (20.50–27.00)	30.90 (21.00–35.30)	24.80 (23.20–33.00)	20.45 (17.30–24.30)	0.178
WHR	0.87 (0.83–0.93)	0.98 (0.84–1.00)	0.89 (0.87–0.97)	0.81 (0.79–0.90)	0.168
SMM (kg)	25.90 (23.00–29.00)	24.80 (22.30–26.40)	22.20 (21.80–27.90)	23.05 (20.20–23.40)	0.335
FAT (kg)	21.60 (12.90–29.40)	36.70 (18.80–45.70)	21.90 (20.80–41.50)	14.00 (11.90–23.40)	0.245
TBW (%)	34.10 (31.00–38.00)	32.70 (29.80–34.60)	29.70 (29.10–36.90)	30.90 (27.70–31.10)	0.311
FFM (%)	46.60 (42.30–52.00)	44.70 (40.70–47.40)	40.70 (39.80–50.50)	42.10 (37.90–42.60)	0.331
PBF (%)	31.70 (23.40–37.80)	43.70 (30.10–52.20)	36.30 (30.00–42.60)	25.90 (22.10–36.70)	0.180
VFA (cm^2^)	9.00 (5.00–14.00)	19.00 (7.00–22.00)	10.00 (8.00–20.00)	5.00 (4.00–12.00)	0.233
BMR (kcal)	1377 (1285–1492)	1336 (1249–1393)	1249 (1230–1460)	1279.5 (1188–1290)	0.318

Legend: PCOS, polycystic ovary syndrome; BMI, body mass index; WHR, waist hip ratio; SMM, skeletal muscle mass; FAT, fat mass; TBW, total body water; FFM, fat-free mass; PBF, percent of body fat; VFA, visceral fat area; BMR, basal metabolic rate.

**Table 3:** Correlation coefficient between SHBG or fTest concentration and selected body composition parameters in the group of women with PCOS.

Parameters	SHBG	fTest
BMI (kg/m^2^)	−0.68; 0.000	0.47; 0.000
WHR	−0.68; 0.000	0.45; 0.000
SMM (kg)	−0.47; 0.000	0.37; 0.004
FAT (kg)	−0.63; 0.000	0.42; 0.001
TBW (%)	−0.47; 0.000	0.38; 0.004
FFM (%)	−0.47; 0.000	0.38; 0.004
PBF (%)	−0.57; 0.000	0.37; 0.000
VFA (cm^2^)	−0.61; 0.000	0.41; 0.001
BMR (kcal)	−0.47; 0.000	0.38; 0.005

Legend: PCOS, polycystic ovary syndrome; SHBG, sex hormone-binding globulin; fTest, free testosterone; BMI, body mass index; WHR, waist hip ratio; SMM, skeletal muscle mass; FAT, fat mass; TBW, total body water; FFM, fat-free mass; PBF, percent of body fat; VFA, visceral fat area; BMR, basal metabolic rate.

**Table 4:** Logistic regression of the state variable of study group vs. control group.

Model 1		Model 2		Model 3		Model 4	
Variables	OR (95% CI) *p*	Variables	OR (95% CI) *p*	Variables	OR (95% CI) *p*	Variables	OR (95% CI) *p*
AMH (ng/mL)	2.085 (1.451–2.997) <0.001	AMH (ng/mL)	2.057 (1.445–2.929) <0.001	AMH (ng/mL)	2.064 (1.442–2.952) <0.001	AMH (ng/mL)	2.085 (1.451–2.997) <0.001
BMI (kg/m^2^)	1.198 (1.036–1.386) 0.015	PBF (%)	1.079 (1.002–1.162) 0.043	VFA (cm^2^)	1.141 (1.012–1.287) 0.031	BMI (kg/m^2^)	1.198 (1.036–1.386) 0.015

Model 1 adjusted to: all hormones under fasting condition and BMI, Model 2 adjusted to all hormones under fasting condition and PBF, Model 3 adjusted to all hormones under fasting condition and VFA, Model 4 adjusted to all hormone under fasting condition and BMI, PBF, VFA. Legend: AMH, Anti-Müllerian hormone; BMI, body mass index; PBF, percent of body fat; VFA, visceral fat area; OR, odds ratio; CI, confidence interval.

## Data Availability

The data presented in this study are available upon request from the corresponding author.
